# Relationship between Aldosterone and the Metabolic Syndrome in Patients with Obstructive Sleep Apnea Hypopnea Syndrome: Effect of Continuous Positive Airway Pressure Treatment

**DOI:** 10.1371/journal.pone.0084362

**Published:** 2014-01-20

**Authors:** Antonia Barceló, Javier Piérola, Cristina Esquinas, Mónica de la Peña, Meritxell Arqué, Alberto Alonso-Fernández, Josep Miquel Bauçà, Juan Robles, Bernardino Barceló, Ferran Barbé

**Affiliations:** 1 Servei d'Anàlisis Cliniques, Hospital Universitari Son Espases, Palma de Mallorca; Servei de Pneumologia, Spain; 2 Pneumologia, Hospital Universitari Son Espases, Palma de Mallorca; Servei de Pneumologia, Spain; 3 Unitat d'Investigació Hospital Universitari Son Espases, Palma de Mallorca; Servei de Pneumologia, Spain; 4 Hospital Arnau de Vilanova/Santa Maria, Lleida, Spain; 5 Centro de Investigación Biomédica en Red de Enfermedades Respiratorias (CIBERES), Mallorca, Illes Balears, Spain; University of Pittsburgh, United States of America

## Abstract

**Background:**

Metabolic syndrome (MS) occurs frequently in patients with obstructive sleep apnea-hypopnea syndrome (OSAHS). We hypothesized that aldosterone levels are elevated in OSAHS and associated with the presence of MS.

**Methods:**

We studied 66 patients with OSAHS (33 with MS and 33 without MS) and 35 controls. The occurrence of the MS was analyzed according to the National Cholesterol Education Program Adult Treatment Panel III (NCEP ATP III) clinical criteria. Measurements of plasma renin activity (PRA), aldosterone, aldosterone:PRA ratio, creatinine, glucose, triglycerides, cholesterol and HDL cholesterol were obtained at baseline and after CPAP treatment.

**Results:**

Aldosterone levels were associated with the severity of OSAHS and higher than controls (p = 0.046). Significant differences in aldosterone levels were detected between OSAHS patients with and without MS (p = 0.041). A significant reduction was observed in the aldosterone levels in patients under CPAP treatment (p = 0.012).

**Conclusion:**

This study shows that aldosterone levels are elevated in OSAHS in comparison to controls, and that CPAP therapy reduces aldosterone levels. It also shows that aldosterone levels are associated with the presence of metabolic syndrome, suggesting that aldosterone excess might predispose or aggravate the metabolic and cardiovascular complications of OSAHS.

**Trial registration:**

The study is not a randomized controlled trial and was not registered.

**What is the key question?**

Are aldosterone levels elevated in patients with obstructive sleep apnea-hypopnea syndrome (OSAHS), and associated with the presence of metabolic syndrome (MS)?

**What is the bottom line?**

Current data indicate that OSAHS is highly associated with MS. The underlying mechanistic links between OSAHS and MS are not well delineated to date.

There is a close relationship between the Renin-Angiotensin-Aldosterone system and hypertension, and recent evidence involves aldosterone in the pathogenesis of MS.

**Why read on?**

These findings show that aldosterone levels are elevated in OSAHS in comparison to controls, and continuous positive airway pressure (CPAP) therapy reduces aldosterone levels. They also show that aldosterone levels are associated with the presence of metabolic syndrome, suggesting a potential role of aldosterone excess in the development of metabolic and cardiovascular complications in patients with OSAHS.

**What is the bottom line?**

Current data indicate that OSAHS is highly associated with MS. The underlying mechanistic links between OSAHS and MS are not well delineated to date.

There is a close relationship between the Renin-Angiotensin-Aldosterone system and hypertension, and recent evidence involves aldosterone in the pathogenesis of MS.

**Why read on?**

These findings show that aldosterone levels are elevated in OSAHS in comparison to controls, and continuous positive airway pressure (CPAP) therapy reduces aldosterone levels. They also show that aldosterone levels are associated with the presence of metabolic syndrome, suggesting a potential role of aldosterone excess in the development of metabolic and cardiovascular complications in patients with OSAHS.

## Introduction

Current data indicate that obstructive sleep apnea-hypopnea syndrome (OSAHS) is highly associated with the metabolic syndrome (MS) [1]–[3] The underlying mechanistic links between OSAHS and the metabolic syndrome have not been well delineated.

There is a close relationship between the Renin-Angiotensin-Aldosterone (RAS) system and hypertension [4]–[6]. Furthermore, recent evidence involves aldosterone in the pathogenesis of metabolic syndrome [7],[8]. Findings from observational studies demonstrated that high aldosterone levels are associated with impaired pancreatic β-cell function and insulin resistance and long-term clinical trials of antihypertensive agents have shown that direct inhibitors of the RAS system (angiotensin converting enzyme inhibitors and angiotensin receptor blockers) significantly improved insulin sensitivity and reduced the risk of incident diabetes[9]. Several cross-sectional studies have demonstrated that higher aldosterone levels are associated with a greater prevalence of MS and components of MS[10]–[13]. In the Framingham Offspring study aldosterone was found to correlate positively with both the development of the MS and an increase in systolic blood pressure, indicating that aldosterone would predict the onset of hypertension and the MS[14].

It has been suggested that activation of the RAS system, in particular aldosterone excess, may play a pathophysiological role in the relation between OSAHS and hypertension[15],[16]. The possibility that this activation may contribute to the development of the metabolic syndrome in OSAHS is unknown. We hypothesized that hyperaldosteronism is highly prevalent in OSAHS and also associated with the presence of MS. To test this hypothesis, we evaluated the plasma aldosterone concentrations (PAC) and plasma aldosterone: renin activity (PRA) ratio in patients with OSAHS (with and without MS) and controls without MS. Patients were re-examined after 12 months of effective treatment with continuous positive airway pressure (CPAP).

## Methods

### Subjects and ethics

We included in the study 66 male patients with OSAHS (33 with MS and 33 without MS). Patients were matched for age (±5 years) and BMI (±3 Kg.m^−2^). As a reference group, we studied 35 controls without MS. Participants were recruited from subjects who attended our sleep unit. No participant suffered from any other chronic disease (diabetes, chronic obstructive pulmonary disease (COPD), liver cirrhosis, thyroid dysfunction, rheumatoid arthritis, chronic renal failure and/or psychiatric disorders). In addition, the enrolled participants were taking no antihypertensive medications and there were no differences between the number of patients and controls taking hypoglycemic, hypolipemiant and/or anti-inflammatory agents.

Each patient was studied at diagnosis and after effective treatment with CPAP (REM Star, Respironics ®, Murrysville, PA, USA) during 12 months. Fifteen patients who did not use the device for a minimum of 4 h/night or did not return for the follow-up visit at 12 months were excluded from the follow-up analysis. The study was approved by the Ethics Committee of our institution, “Comitè Ètic d'Investigació Clínica Illes Balears”, and all participants signed their consent after being fully informed of its goal and characteristics.

### Measurements and definitions

The diagnosis of OSAHS was established by full polysomnography (E-Series Compumedics, Abbotsford, Australia), which included recording of oronasal flow, thoracoabdominal movements, electrocardiography, submental and pretibial electromyography, electrooculography, electroencefalography and trancutaneous measurement of arterial oxygen saturation. Apnea was defined by the absence of airflow for more than 10 seconds. Hypopnea was defined as any airflow reduction that last more than 10 seconds and resulted in arousal or oxygen desaturation. We considered desaturation a decrease in SaO_2_ greater than 4%. The apnea-hypopnea index (AHI) was defined as the sum of the number of apneas plus hypopneas per hour of sleep. The case or control status was defined by the AHI threshold of 10 or greater. Excessive daytime sleepiness (EDS) was subjectively quantified by the Epworth sleepiness scale (ESS).

The occurrence of the MS was analyzed according to the National Cholesterol Education Program Adult Treatment Panel III (NCEP ATP III) clinical criteria: (1) waist circumference ≥94 cm in men and ≥88 cm in women, (2) fasting glucose ≥100 mg/dL or patient on specific drug treatment, (3) triglycerides ≥150 mg/dL or patient on specific treatment, (4) HDL cholesterol (HDLc) <40 mg/dL in men and <50 mg/dL in women or patient on specific drug treatment, (5) systolic blood pressure (SBP) ≥130 mmHg or diastolic blood pressure(DBP) ≥85 mmHg or patient on specific drug treatment. The MS was diagnosed if 3 of these 5 factors were present.

After fasting overnight, venous blood samples were obtained between 8 and 10 am. Blood was centrifuged and plasma and serum were immediately separated in aliquots and stored at –80°C until analysis.

Glucose, triglycerides, total cholesterol, HDL cholesterol (HDLc), and creatinine were determined by standard enzymatic methods on a Hitachi Modular analyzer (Roche Diagnostics, Indianapolis, USA). Plasma renin activity (PRA) and serum aldosterone were measured by radioimmunoassay methods (DPC Biermann, Bad Nauheim, Germany). Hyperaldosteronism was defined as an aldosterone:PRA ratio at least 30 (ng/dL/ng/mL/h) and/or a plasma aldosterone more than 20 ng/dL.

### Statistical analysis

Results are presented as percentages or mean ± standard deviations. Comparisons between groups were performed by Kruskal Wallis or ANOVA test for continuous variables and χ^2^ test for categorical variables.

The effects of CPAP therapy were analyzed using paired t-tests (Wilcoxon or McNemar test).

Correlations between variables were explored using the Spearman-rank test.

In order to evaluate the relationships between OSAHS, aldosterone and the metabolic syndrome, logistic regression models were calculated with MS and its components as dependent and aldosterone as independent variables after adjusting for age and BMI.

A p value lower than 0.05 was considered significant. The SPSS v.18 software was used for all analyses.

## Results

Characteristics of the study population are summarized in Table 1. By design, age and BMI were similar in patients with and without MS.

**Table 1:** Subject characteristics.

	OSAHS with MS (n = 33)	OSAHS without MS (n = 33)	Controls (n = 35)
Age (years)	52±8**	49±9**	42±15
BMI (Kg.m ^−2^)	32±3**	29±4**	26±4
AHI (hour ^−1^)	53±24**	45±18**	5±2
Mean Sat O_2_(%)	92±3**	93±2**	96±2
Minimal Sat O_2_(%)	78±9**	82±6**	90±3
Epworth scale	10±4	9±4	9±3
Arousal index	64±22**	52±16**	19±12
Waist circumference (cm)	110±8**	104±9**	94±11
Glucose (mg/dL)	117±22*	98±10	95±18
Triglycerides (mg/dL)	236±61*	127±62	112±46
HDLc (mg/dL)	46±9	55±12	52±10
SBP (mmHg)	141±15*	125±15*	118±13
DBP (mmHg)	88±10*	79±10*	72±8
Creatinine (mg/dL)	0.94±0.15	0.96±0.11	0.94±0.12
PRA (ng/mL/h)	1.3±1.2	1.9±1.1	1.3±1.1
Aldosterone (ng/dL)	17.8±9.4*	14.8±9.7*	13.1±7.4
Aldosterone/PRA ratio	21±15	19±17	17±15
Hyperaldosteronism (n,%)	(9, 28%)*	(3,10%)	(2, 6%)

p<0.01, * p<0.05 versus controls,

p<0.05 versus OSAHS without MS.

Aldosterone levels were significantly higher in OSAHS patients than in subjects without OSAHS (p = 0.046). Furthermore, significant differences in aldosterone levels were detected between OSAHS patients with and without MS (p = 0.041).

**Figure 1:**
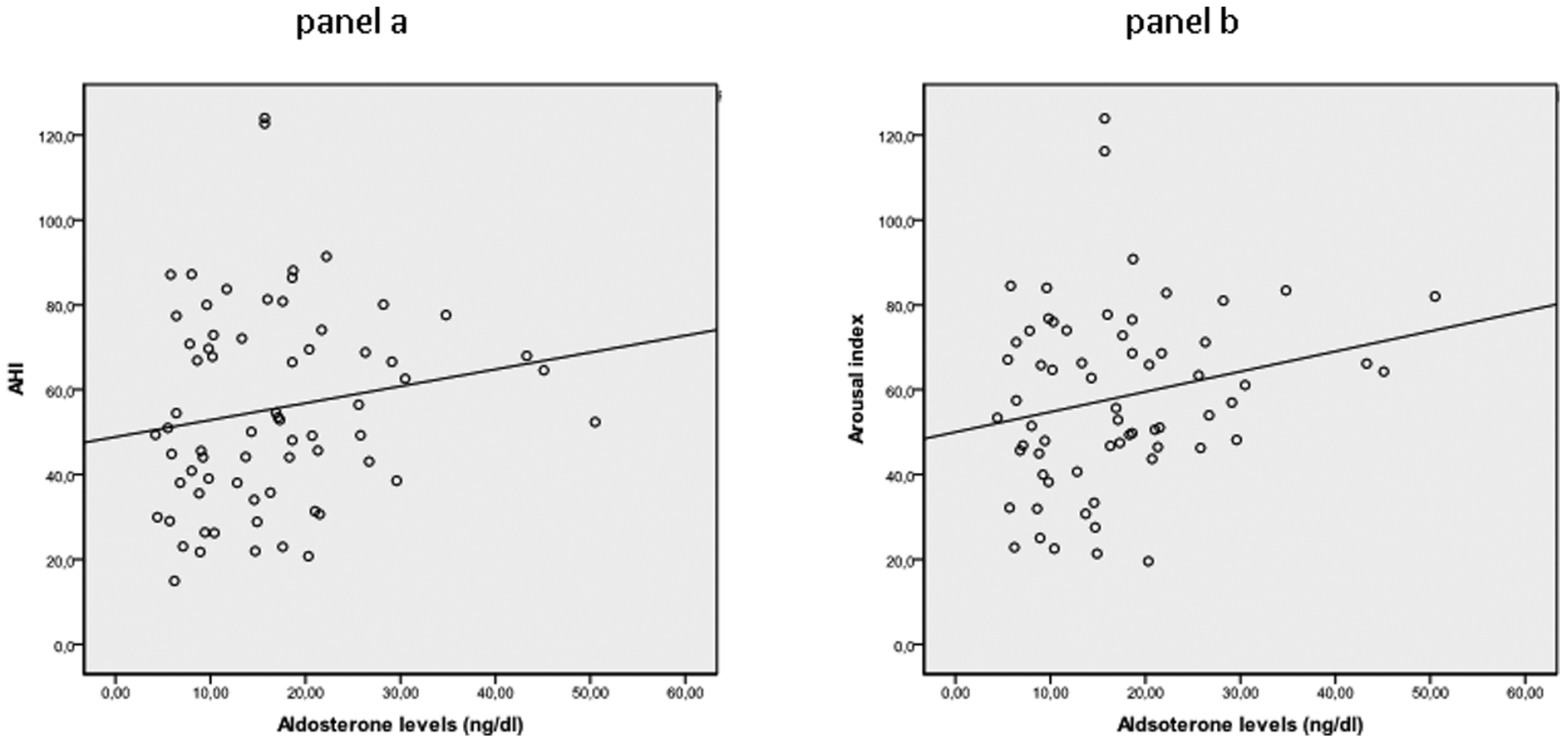
Relationship between aldosterone and apnea-hypopnea index (panel a) and arousal index (panel b) in the OSAHS population studied.

**Figure 2:**
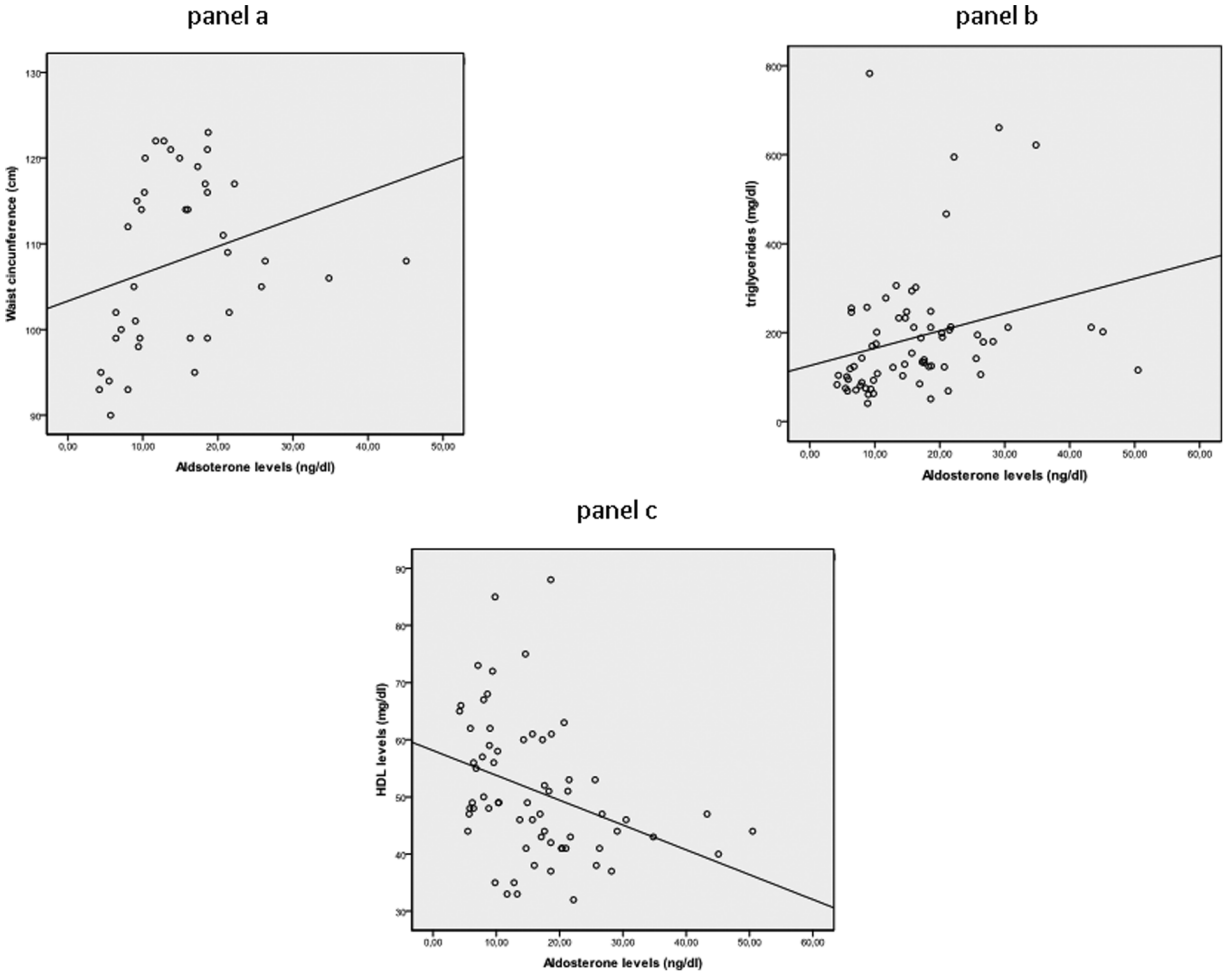
Relationship between aldosterone and waist circumference (panel a), triglycerides (panel b), and HDL cholesterol (panel c) in the OSAHS population studied.

In the OSAHS group, aldosterone levels were significantly related to AHI (r = 0.225, p = 0.016) and to arousal index (r = 0.269, p = 0.05), (Figure 1, panels a and b, respectively). Likewise, aldosterone levels were also significantly related to waist circumference (r = 0.431, p = 0.006), triglyceride levels (r = 0.395, p = 0.001) and HDL cholesterol levels (r = 0.439, p = 0.001), (Figure 2, panels a, b, c)

Aldosterone levels tended to be higher in hypertensive patients (19.2±12.2 ng/dL, n = 14) than in normotensive patients (15.3±8.6 ng/dL), although differences did not reach statistical significance (p = 0.142).

**Figure 3:**
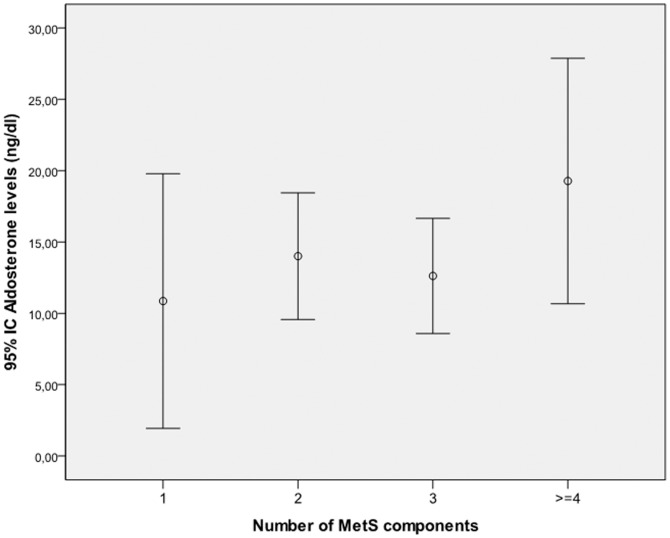
Aldosterone levels by number of metabolic risk factors.

In a multiple regression analysis, a meaningful association between aldosterone levels and the presence of metabolic syndrome was found. In addition, aldosterone levels were associated with the presence of elevated blood pressure (≥130/85 mmHg) and elevated triglyceride levels (≥150 mg/dL), according to criteria for the MS. Aldosterone levels tended to increase with an increasing number of MS risk factors (p = 0.08), (Figure 3).

Four patients with MS showed glucose levels higher than 126 mg/dL (threshold value for defining diabetes), although their exclusion would not change initial results.

The results of the metabolic parameters at baseline and at the follow-up examination are shown in table 2. A statistically significant reduction was observed in the levels of aldosterone in patients under CPAP treatment, without significant changes in PRA and aldosterone/PRA ratio. They also manifested a significant increase in HDLc cholesterol compared to baseline despite similar BMI. There were no significant variations in waist circumference, SBP, DBP, glucose or triglyceride levels.

**Table 2:** Changes in components of the metabolic syndrome and in aldosterone, PRA and aldosterone/PRA ratio after 12 month of CPAP treatment (n = 51).

	Baseline	Follow-up	p value
BMI (Kg.m ^−2^)	30.5±4.5	30.9±4.5	0.191
Waist circumference (cm)	106.1	107.5	0.706
Glucose (mg/dL)	108±22	106±33	0.246
Triglycerides (mg/dL)	193±62	179±75	0.288
HDLc (mg/dL)	51±10	54±11	0.022
SBP (mmHg)	134±16	138±20	0.252
DBP (mmHg)	83±11	81±10	0.311
Creatinine (mg/dL)	0.95±0.15	0.94±0.15	0.855
PRA (ng/mL/h)	1.4±1.1	1.3±0.9	0.765
Aldosterone (ng/dL)	16.7±8.7	12.6±9.4	0.012
Aldosterone/PRA ratio	19±15	17±13	0.274
Hyperaldosteronism (n,%)	9, 19%	2, 4%	0.120

## Discussion

The results of this study show that aldosterone levels are associated to the severity of OSAHS and that CPAP therapy reduces aldosterone levels. They also show that aldosterone levels are associated with the presence of metabolic syndrome, suggesting that aldosterone excess might predispose or aggravate the metabolic and cardiovascular complications of OSAHS.

The relation between OSAHS and the renin-angiotensin-aldosterone system (RAS) is complex and data are not uniform. Aldosterone is synthesized by the adrenal gland in response to angiotensin II, potassium and adrenocorticotrophin hormone (ACTH). In addition, obesity is associated with increased levels of the circulating RAS components [24]–[28]. The adipose tissue is an important source of the renin substrate (angiotensinogen), but other mediators can stimulate aldosterone synthesis independently of angiotensin II, as well [8],[29]. Experimental studies have shown that adipocytes may release adipokines and free fatty acids that could either directly or indirectly stimulate aldosterone secretion by adrenocortical cells [30]–[32]. In our study, aldosterone levels were higher in OSAHS patients than in controls. We also found a significant correlation between aldosterone levels (but not PRA) and AHI and arousal index, suggesting that sleep fragmentation and repetitive arousals may influence aldosterone secretion in patients with OSAHS. Aldosterone could be elevated by a repeated stresses-based mechanism, which could impact ACTH release and/or by additional RAS-independent stimulus. This interpretation is further supported by the observation that treatment of OSAHS with CPAP reduces aldosterone levels, without modifying BMI and PRA levels.

Recent studies involve aldosterone in the pathogenesis of the metabolic syndrome [33],[34]. Elevated aldosterone levels have been pointed to lead not only to sodium retention and volume expansion, but also to increased inflammation and oxidative stress, which in turn promote insulin resistance, impaired pancreatic β cell function, endothelial dysfunction and hypertension [35],[36]. There is also evidence that aldosterone may worsen preexisting alterations in glucose and lipid metabolism [37],[38]. In addition, a recent study demonstrated that in patients with hypertension, plasma aldosterone levels were higher in the group with MS in spite of no differences in plasma renin activity between the groups with or without MS [10].

Prevalence of the MS is higher in OSAHS patients than in the general population, but the biological mechanisms underlying the link between OSAHS and MS are not yet fully understood [3]. In this study, we evaluated whether aldosterone may play a role in the development of the MS in OSAHS.

Aldosterone levels were higher in the OSAHS group with MS and in a multivariate-adjusted analysis aldosterone levels were significantly associated with the presence of metabolic syndrome, elevated triglyceride levels and elevated blood pressure. OSAHS patients with MS presented higher levels of glucose than patients without MS, but the association between aldosterone and glucose levels failed to reach statistical significance. Experimental and clinical evidence support a pathophysiologic link between OSAHS, beta cell function and glucose metabolism [3],[38]. Moreover, several cross-sectional studies have reported direct correlations between aldosterone and insulin resistance [27],[30]. Despite these evidences, we found that aldosterone was associated with the MS, even after adjusting for all of its single components, such as high glucose levels, which would indicate that the clustering of these factors per se is associated with aldosterone. The importance of aldosterone in the incidence of metabolic syndrome is supported by follow-up sub analyses from the Framingham Offspring Study. In a recent investigation on this group, among 8 biomarkers representing distinct biological pathways to the incidence of MS only plasminogen activator inhibitor-1 (PAI-1) and aldosterone were significantly associated with the incidence of metabolic syndrome, relations that remained robust after adjustment for insulin resistance (HOMA-IR). In our study, aldosterone levels were significantly associated with the presence of metabolic syndrome. Furthermore, aldosterone levels tended to increase with an increasing number of MS risk factors, suggesting that aldosterone excess may predispose or aggravate the development of metabolic syndrome. At the individual level, future research should focus on a better understanding the associations of aldosterone with separate components of MS, including glucose and lipid metabolism and hypertension.

Recent studies have suggested that aldosterone excess may play a role in the relation between hypertension and OSAHS [15],[17],[18]. The mechanisms linking OSAHS and hyperaldosteronism are not fully elucidated and it is not clear if whether aldosterone secretion is cause or consequence of OSAHS [19]. OSAHS may lead to increased aldosterone release via stimulation of the RAS, which in turn may might contribute to OSAHS through excess fluid retention and upper airway narrowing [16],[20]. A recent study demonstrated that treatment with spironolactone could reduces OSAHS severity in patients with resistant hypertension [21]. In another study evaluating the effects of CPAP, a reduction in aldosterone levels after 3 months of CPAP treatment has been detected [22]. In contrast, Svatikova et al found that patients with OSAHS without co-existing co morbidities have aldosterone and renin levels similar to healthy subjects [23]. Despite the fact that our results revealed a significant association between aldosterone and elevated blood pressure according to criteria for the MS, there were no differences in aldosterone levels between normotensive and hypertensive patients, which may have different reasons. First, we carefully excluded patients taking antihypertensive medications. The excluded patients could represent the majority of patients with elevated blood pressure. This introduces a selection in the study population and reduces statistical power. Second, the effects of the medications may be divergent (suppress or increase) on aldosterone levels and renin activity, and methodological differences may contribute to variability between studies.

### Limitations

Some potential confounding factors, such as nutritional status, physical activity or the interaction between genetic variants, were not taken into account in our analysis.

Participants were all male. This fact limits the generalizability of the results to the whole population. We think that future randomized studies including these measurements are needed to determine the impact of all these observations on metabolic dysfunction of OSAHS patients.

## Conclusions

This study shows that aldosterone levels are elevated in OSAHS in comparison to controls, and that CPAP therapy reduces aldosterone levels. It also shows that aldosterone levels are associated with the presence of metabolic syndrome, suggesting that aldosterone excess might predispose or aggravate the metabolic and cardiovascular complications of OSAHS.
